# Bedside ultrasonography for rapid detection of splenic abscess in melioidosis

**DOI:** 10.1590/0037-8682-0097-2023

**Published:** 2023-06-02

**Authors:** Chee Yik Chang

**Affiliations:** 1 Medical Department, Hospital Kapit, Sarawak, Malaysia.

**FIGURE 1:**
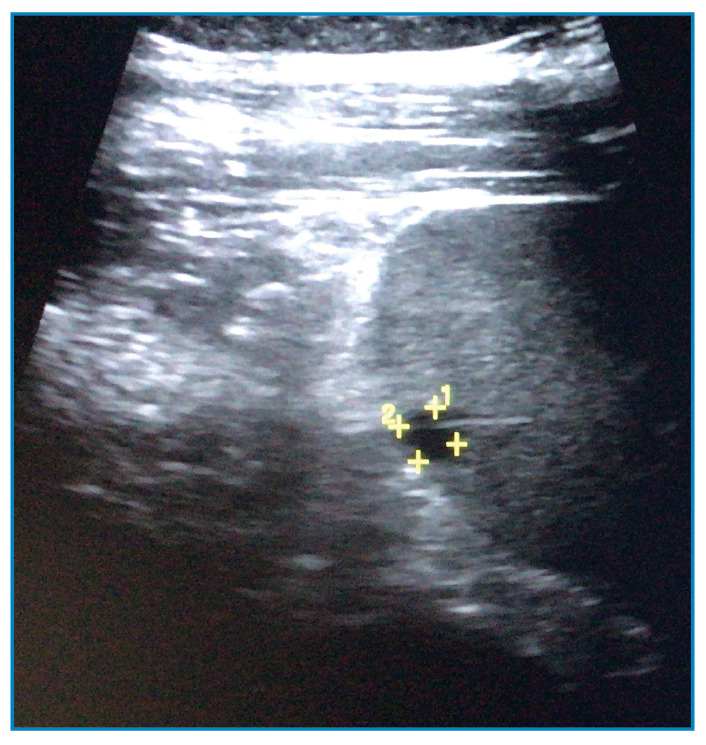
Abdominal ultrasound scan showing a hypoechoic splenic lesion measuring 0.8 cm × 0.8 cm, indicative of an abscess.

A 35-year-old farmer presented with a 3-week history of intermittent fever and malaise. Physical examination showed stable vital signs and no other abnormalities. Bedside ultrasonography performed for investigation of pyrexia of unknown origin revealed a small splenic microabscess measuring 0.8 cm × 0.8 cm (Figure 1). Chest radiography and transthoracic echocardiography findings were normal. The patient was empirically treated for melioidosis using intravenous ceftazidime, and blood culture results were positive for *Burkholderia pseudomallei,* which confirmed the diagnosis. The patient received 4-week treatment with intravenous ceftazidime, followed by a 3-month course of oral trimethoprim-sulfamethoxazole, which led to resolution of the splenic abscess. 

Melioidosis is a potentially fatal disease caused by the *B. pseudomallei* bacterium, endemic to Southeast Asia and Northern Australia. Melioidosis is commonly complicated by the development of liver or splenic abscesses, which can be difficult to diagnose because of nonspecific symptoms^1^. The Darwin melioidosis study reported that internal organ abscesses, including prostatic (20%), splenic (5%), and liver abscesses (3%) were common in this patient population^2^. Bedside ultrasonography is noninvasive, readily available, and has high sensitivity and specificity; therefore, this modality has emerged as a valuable tool for diagnosis of melioidosis-induced liver/splenic abscesses. A recent study in Laos reported that abscesses had a positive predictive value of 93% (88-96%) for melioidosis^3^. Therefore, bedside ultrasonography is a useful method for detection of visceral abscesses in febrile patients in endemic areas and facilitates initiation of prompt empirical antibiotic therapy for melioidosis while awaiting confirmation of culture results.
